# A major genetic locus controlling natural *Plasmodium falciparum *infection is shared by East and West African *Anopheles gambiae*

**DOI:** 10.1186/1475-2875-6-87

**Published:** 2007-07-06

**Authors:** Michelle M Riehle, Kyriacos Markianos, Louis Lambrechts, Ai Xia, Igor Sharakhov, Jacob C Koella, Kenneth D Vernick

**Affiliations:** 1 Microbial and Plant Genomics Institute and Department of Microbiology, University of Minnesota, St. Paul, MN 55108, USA; 2 Program in Genomics, Department of Medicine, Children's Hospital Boston, Harvard Medical School, Boston, MA 02115, USA; 3 Department of Entomology, University of California, Davis, CA 95616, USA; 4 Department of Entomology, Virginia Tech University, Blacksburg, VA 24061, USA; 5 Division of Biology, Imperial College London, Ascot, Berkshire SL5 7PY, UK

## Abstract

**Background:**

Genetic linkage mapping identified a region of chromosome 2L in the *Anopheles gambiae *genome that exerts major control over natural infection by *Plasmodium falciparum*. This 2L *Plasmodium*-resistance interval was mapped in mosquitoes from a natural population in Mali, West Africa, and controls the numbers of *P. falciparum *oocysts that develop on the vector midgut. An important question is whether genetic variation with respect to *Plasmodium*-resistance exists across Africa, and if so whether the same or multiple geographically distinct resistance mechanisms are responsible for the trait.

**Methods:**

To identify *P falciparum *resistance loci in pedigrees generated and infected in Kenya, East Africa, 28 microsatellite loci were typed across the mosquito genome. Genetic linkage mapping was used to detect significant linkage between genotype and numbers of midgut oocysts surviving to 7–8 days post-infection.

**Results:**

A major malaria-control locus was identified on chromosome 2L in East African mosquitoes, in the same apparent position originally identified from the West African population. Presence of this resistance locus explains 75% of parasite free mosquitoes. The Kenyan resistance locus is named EA_Pfin1 (East Africa_ *Plasmodium falciparum *Infection Intensity).

**Conclusion:**

Detection of a malaria-control locus at the same chromosomal location in both East and West African mosquitoes indicates that, to the level of genetic resolution of the analysis, the same mechanism of *Plasmodium*-resistance, or a mechanism controlled by the same genomic region, is found across Africa, and thus probably operates in *A. gambiae *throughout its entire range.

## Background

The mosquito *Anopheles gambiae *is the major African vector of human malaria caused by *Plasmodium falciparum*. More than 300 million cases of acute malaria occur globally each year resulting in over one million deaths with greater than 90% of these deaths occurring in children from Sub-Saharan Africa [1]. In malaria endemic countries where the poorest sections of society are most affected, inadequate public health initiatives and the selection of insecticide resistance hinder the efforts to control malaria transmission. Recent proposals to curb the malaria burden involve more technological approaches including the generation and introduction of transgenic mosquitoes [2]. However, all approaches have their own logistical, political and ethical limitations.

Here the genetics of natural vector resistance to *P. falciparum *is dissected, with the ultimate goal to utilize successful existing natural *Plasmodium *resistance mechanisms for control of human malaria transmission. Knowledge of the prevalence, strength and genomic location of loci that control mosquito resistance to malaria parasites in vector populations could aid future efforts to control parasite transmission. Genetically resistant and susceptible mosquitoes exist in nature, and phenotypic variation can segregate as a simple Mendelian trait [3]. A recent comprehensive study reported the prevalence, strength and genomic location of natural *P. falciparum *resistance loci in *A. gambiae *in Mali, West Africa [4]. The loci with the strongest phenotypic effect on parasite numbers clustered to form a *Plasmodium*-resistance island (PRI) on chromosome 2L. It would be valuable to know whether the genetic mechanism of the PRI identified in Mali is specific to that population, suggesting that there might be many geographically local *A. gambiae *resistance mechanisms across Africa, or conversely whether the mechanism identified by the PRI exists in *A. gambiae *throughout Africa. A major barrier to gene flow in *A. gambiae *occurs between West and East African populations [5], and thus comparing these populations should offer the most stringent test of the geographic generality of genetic loci controlling resistance mechanisms.

## Methods

The source of the pedigrees was previously reported [6]. Briefly, isofemale pedigrees were initiated from a laboratory colony (established in Mbita, Kenya in 2001, approximately three years prior to use in this study) and raised to the F1 generation at which point they were challenged on gametocyte containing blood. The same pedigrees were previously used to investigate the role of genotype by genotype interactions in controlling the parasite load of vector mosquitoes [6]. Thus the parasite challenge was heterogeneous: each F1 pedigree was split into 3–5 groups that were each fed on independent natural gametocytemic blood samples. Unfed and partially fed females were excluded from further analysis. Seven to eight days after the infective blood meal, mosquito midguts were dissected and stained with 2% mercurochrome, and the numbers of oocysts were counted by light microscopy. Mosquito carcasses were saved for later DNA extraction.

**Figure 1:**
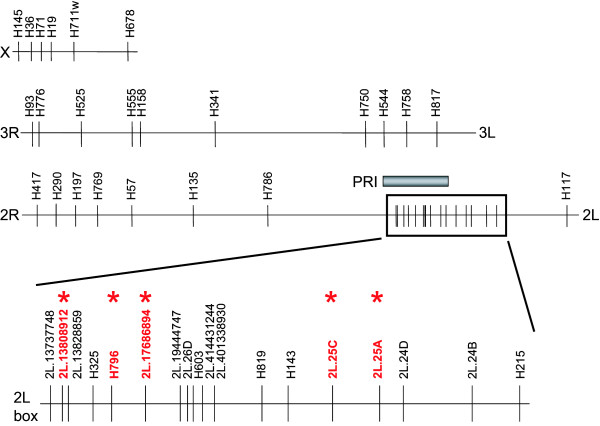
**Physical positions of microsatellite markers used in genetic mapping**. The three chromosomes are indicated as horizontal lines (from top: X, 3, 2), and positions of marker loci are vertical lines labeled with the marker name. Note the high density of markers on chromosome 2L (boxed section expanded in bottom horizontal line, labeled 2L box), which were used to map at greater density once an initial linkage signal was detected. Dense markers were necessary so that the most informative markers (i.e., those with the greatest numbers of alleles segregating) could be used in the analysis. The five clustered marker loci giving significant linkage (linkage data shown in Table 1) are depicted in bold red type and are starred. For comparison, the extent of the *Plasmodium*-resistance island mapped in West African pedigrees [4] is indicated by the filled bar above chromosome 2L (labeled PRI). Chromosome 2 is shown in the 2La inverted (2La/a) conformation.

DNA was isolated from mosquitoes using Qiagen DNeasy (Qiagen, CA, USA). Microsatellite genotyping was performed multiplexed as described previously [4]. Fragments were separated on an ABI3100 Genetic Analyzer and sized using GeneMapper Version 3.5 (both from Applied Biosystems, CA, USA). All automated genotype calls were visually verified. Mapping was carried out in those pedigrees deemed potentially informative using previously described guidelines; ≥20 females with ≥30% infection prevalence [4]. Six pedigrees, named 57, 92, 141, 142, 146 and 1102 met the typing criteria. Twenty-eight microsatellite loci were screened in these informative pedigrees and as many as 15 additional loci were typed in some, but not all pedigrees (Figure 1). Many of these additional markers were added to increase the signal resolution around marker H325. All primer sequences are available elsewhere [4,7] except 2L.24D left 5' TGCCAATCAATCAGTGTGCT 3' and right 5' CACTAGCAACGGCACTACCC 3' and 2L.26D left 5' GAAGTGGAAGAACACGCTCA and right 5' CACACGATTGCAGATGAGTT 3'. Physical positions given are based on Ensembl *A. gambiae *genome sequence version 39, converted to the inverted 2La/a form by use of inversion breakpoint coordinates from [8].

Linkage analysis methods are described in detail elsewhere [3]. Despite the fact that each pedigree was divided into groups that were fed on different gametocyte carriers, in the current study all individuals within a pedigree were first analysed together. In one pedigree yielding a linkage signal, the infections from different parasite isolates were also analysed individually. In brief, at every marker, the pedigree members were split into two samples, using an allele or genotype observed at that marker as the selection criterion. At each locus, the distribution of oocyst numbers for each allele/genotype was compared against the pooled oocyst distribution for all other alleles/genotypes. Permutations were used to establish empirical genome-wide p-values. Wilcoxon-Mann-Whitney (WMW), a nonparametric statistical test, was used to determine statistical significance.

A novel and important aspect of this mapping methodolgy is that it examines each locus in isolation from all other loci and does not require knowledge of relative marker position. Thus, the result is robust to the presence of segregating chromosomal inversions, which are common in *A. gambiae*, and can alter the physical relationship between markers in different individuals within a pedigree. For example, another recent mapping study of *P. falciparum *resistance in other Kenyan pedigrees [9] used a mapping approach in which valid linkage results are dependent on correct knowledge of marker order, but marker order was not empirically determined. That study assumed a marker order [7] that predated release of the *A. gambiae *genome sequence. In fact, marker order in the genome sequence differs markedly from that in [7,9] (e.g., chromosome 2L ref [7,9] order: AG2H46-AG2H197-AG2H786-AG2H135-AG2H603-AG2H787-AG2H325; genome order and current study: AG2H46-AG2H197-AG2H135-AG2H786-AG2H325-AG2H787-AG2H603).

The statistical analysis was run using both the program OUTBRED_LINES as previously described [3] and a new implementation of the same algorithm in the statistical analysis environment R [10], called OUTBRED_LINES-R. The motivation was to avoid use of approximations employed in OUTBRED_LINES and instead use the built-in exact WMW tests. However, very similar results were obtained from OUTBRED_LINES and OUTBRED_LINES-R. Exact p-values from the R implementation OUTBRED_LINES-R are presented throughout the manuscript.

## Results

**Figure 2:**
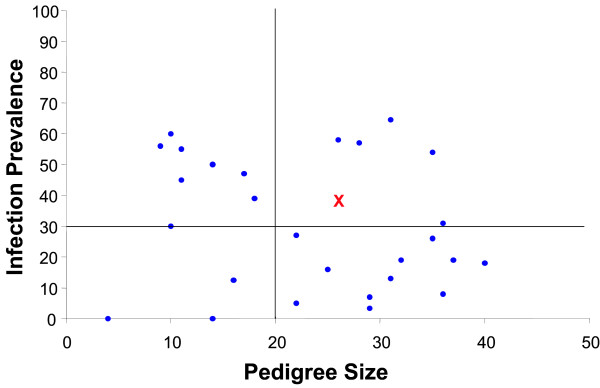
**Pedigree criteria for genetic analysis**. Pedigrees were chosen for genome-wide genotyping and linkage analysis based on unbiased criteria of infection prevalence (the percent of mosquitoes with at least one midgut oocyst) and pedigree size. These criteria, previously applied in the West African pedigree study [4], prioritize the full analysis of the potentially most informative pedigrees. Pedigrees with ≥20 mosquitoes were analysed (actual range 25–36) and ≥30% infection prevalence (actual range, 31–64%). Only 6 of 28 pedigrees met this criterion, shown in the upper right quadrant. Pedigrees falling outside these boundaries are unlikely to yield a detectable genetic signal, even if segregating strong alleles for resistance/susceptibility. The horizontal line inside the graph indicates 30% prevalence and the vertical line indicates a pedigree size of 20. The red x indicates the pedigree that gave a significant linkage signal.

**Figure 3:**
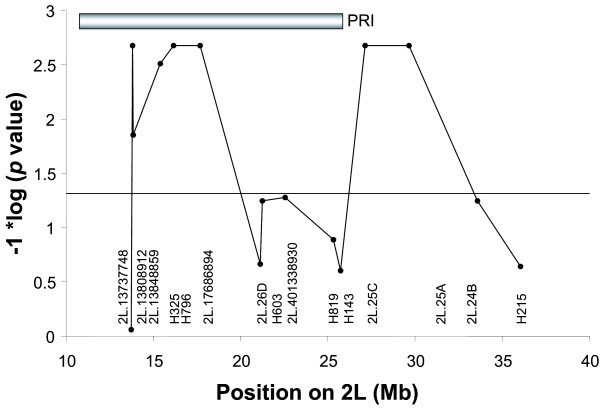
**Mapping of the chromosomal region containing EA_Pfin1**. Nominal *p *values are plotted as a function of physical position. For reference, a value of 1.3 on the transformed y-axis corresponds to a *p *value of 0.05, shown as a horizontal line. The order of markers uses the inverted (2La/a) conformation of the 2L chromosome. Only the most informative markers (≥2 alleles and ≥3 genotypes) are shown. Only markers H796, H17686896, and H25C are fully informative, that is, segregate 4 alleles so that a unique microsatellite allele marks each parental chromosome that entered the pedigree. The dip in the center of the plot is likely due to a lack of marker informativeness.

**Figure 4:**
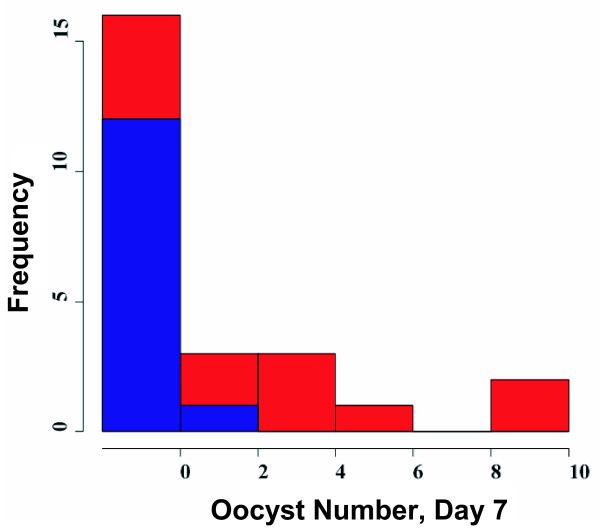
**Oocyst distribution and allele frequency at microsatellite marker H796**. Four alleles are segregating at H796, but only two are informative for infection outcome. Allele 82 (blue) is linked to low oocyst number (average oocyst number 0.08) and allele 84 (red) is linked to high oocyst number (average oocyst number 2.9). Similar histograms can be drawn for the other loci yielding significant linkage with infection outcome.

Six Kenyan pedigrees met the minimum informativeness criteria for genotyping (≥20 mosquitoes surviving to dissection, with infection prevalence ≥ 30%), and these pedigrees were completely genotyped (Figure 2). One of the pedigrees (n = 25, pedigree #57 in [6]) yielded a significant locus for *P. falciparum *resistance (nominal p = 0.002, genome-wide p = 0.040). The locus displays significant linkage to 5 nearby markers (genome wide p <0.05; from left to right; 2L.H13808912, H796, 2L.17686896, 2L.25C, 2L.25A; Figure 1, Table 1). The Kenyan resistance locus is here named EA_Pfin1 (East Africa_ *Plasmodium falciparum *Infection Intensity), to distinguish it from the previously named Pfin1 locus, which it overlaps, identified in the chromosome 2L PRI of West African *A. gambiae *[3,4]. This mapping with greater marker density shows solid statistical support for the EA_Pfin1 resistance locus from multiple genetic markers (Figure 3). The detection of maximum linkage over a large physical region (~16 Mb) is due to the limited number of recombinants expected in such a small pedigree (n = 25). Despite small pedigree size, the EA_Pfin1 locus had high explanatory power for infection phenotype: the marker allele linked to resistance explained 75% of completely uninfected mosquitoes in the pedigree, and the susceptible allele explained 89% of oocyst-containing mosquitoes (Figure 4). The other five informative pedigrees gave no significant linkage signal (discussed below).

**Table 1:** Mapping of the EA_Pfin1 locus.

Marker	Position on 2L	# of Alleles	Allele Linked to High Oocyst #	Allele Linked to Low Oocyst #	Average # of Oocysts with Susceptible Allele	Average # of Oocysts with Resistant Allele
2L.13808912	13.8 MB	3	87	n.d.	2.92	n.d.
H796	16.7 MB	4	84	82	2.92	0.08
2L.17686894	18.3 MB	4	189	180	2.92	0.08
2L.25C	26.6 MB	4	72	132	2.50	0.09
2L.25A	29 MB	3	n.d.	160	n.d.	0.08

Linkage data for the five microsatellite markers significantly linked with *P. falciparum *infection outcome (genome-wide *p *value < 0.05). Physical positions are based on the inverted 2La/a chromosome form. Other markers in this region did not yield significant linkage signal in this pedigree due to a low level of marker informativeness. Because 4 distinct chromosomes were segregating, as indicated by most markers, the two markers with 3 alleles were not fully informative because 1 allele marked 2 chromosomes, thus obscuring the linkage signal from the non-informative cases (labeled n.d., not detected). The nominal and genome-wide *p *values for all markers listed in the table are 0.002 and 0.040, respectively

Careful examination of the EA_Pfin1 pedigree indicates that one of the three parasite isolates used failed to infect any mosquitoes in the pedigree, and infected only five mosquitoes of 76 total assayed across all pedigrees [6]. Due to the lack of infectivity of the parasites in this blood sample for the EA_Pfin1 pedigree, the data from mosquitoes fed on this isolate were removed and the pedigree was reanalysed. Without these six mosquitoes, the remaining 19 mosquitoes still give significant linkage signal at the same microsatellite loci despite the small sample size (Table 2). This result indicates that the linkage signal did not depend solely or disproportionately upon defeat by the mosquitoes of one of the three parasite isolates fed to them. Indeed, even with loss of 24% of the pedigree sample size by removal of the six mosquitoes, the linkage signal was still comparable to that from the entire pedigree. The surviving linkage signal in the 19 mosquitoes was due to mosquitoes in the pedigree whose parasite challenge was divided between two different gametocyte carriers. Analysis of the remaining two groups fed on either gametocyte carrier alone did not yield a linkage signal (not shown).

**Table 2:** Parasite strain transcendence. Comparison of nominal and genome wide p values for linkage at H796 before and after removing mosquitoes fed on a parasite isolate that did not infect any mosquitoes in the pedigree.

**H796**	***p *value**	
	All parasites	Only infective parasites

Nominal	0.002	0.003
Genome-wide	0.040	0.035

To obtain *p *values for only infective parasites, those mosquitoes that fed on the parasite isolate that failed to infect any mosquitoes were removed prior to analysis (n = 6, leaving 19 mosquitoes for analysis)

Thus, the detected linkage signal in the entire pedigree or any subset did not result from a strong protective effect by mosquitoes against any single parasite isolate. Instead, detection of linkage required the larger sample size obtained by combining groups of mosquitoes that fed on at least two different parasite isolates, and consequently the signal must have originated in the pedigree's consistent genetic response to at least two different parasite isolates. Taken together, these results indicate that *Plasmodium *resistance controlled by the *A. gambiae *EA_Pfin1 locus can be independent of and transcend parasite genotype.

## Discussion

A *P. falciparum *resistance locus, EA_Pfin1, is reported in East African *A. gambiae *that resides within the chromosomal *Plasmodium*-resistance Island (PRI) detected in West African *A. gambiae*. The phenotype is also the same: parasite elimination prior to oocyst development. The vast majority (>75%) of mosquitoes in the EA_Pfin1 pedigree carrying the resistance allele completely blocked parasite development, and thus were parasite-free. This is comparable to the phenotypic effect size of resistance loci in the 2L PRI in West Africa [4]. Due to their chromosomal co-localization and similarity of phenotype, EA_Pfin1 and the 2L PRI in West Africa likely control the same underlying *Plasmodium *resistance mechanism.

### Geographic generality of resistance

The geographic generality of resistance mechanisms indicates that the PRI-controlled mechanism of resistance is not a nuance of the pedigrees derived from the West Africa population initially studied. Another recent study in Kenya [9] detected an effect on *P. falciparum *infection outcome, but physical positions of the markers and therefore the mapped loci in that study were not determined and thus it is not possible to resolve whether the effect was controlled by the same genomic region as reported here. Most parsimoniously, a common resistance mechanism across the African continent suggests a relatively ancient origin for natural *P. falciparum *resistance rather than the evolution of distinct resistance mechanisms multiple times. The ancestral PRI-controlled mechanism might have been selected in an *A. gambiae *ancestor by exposure to a non-*Plasmodium *pathogen, or might have newly evolved in *A. gambiae *soon after the advent of the *P. falciparum *host-pathogen system.

### Factors on chromosome 2L control malaria parasite infection

Genetic factors on chromosome 2L have previously been shown to affect malaria infection both in nature [11] and in a laboratory system [12,13]. In addition, the form of the polymorphic 2La inversion is also correlated with adaptation to climate [14], biting and resting behavior [15], and morphology [16]. The previous mapping pedigrees from West Africa were all fixed inverted homozygotes (2La/a) for the 2La inversion [4]. A molecular diagnostic assay designed to determine the orientation of the breakpoints of the 2La inversion [8] indicated that the EA_Pfin1 pedigree in the current study is similarly fixed for the inverted chromosome (data not shown).

Therefore, the data show that at least one strong resistance locus segregates within the inverted form of 2La across Africa. Without homozygous 2La standard form (2La+/+) pedigrees or pedigrees with the 2La inversion segregating (which would include heterozygotes and both homozygotes) the data cannot address whether there is linkage between inversion state and parasite infection loads. Given the lack of any pedigrees in the West African studies fixed for or segregating the 2La+/+ standard form arrangement, and the colony origin and small size of the current East African pedigrees, it is also impossible to know whether this PRI-related mechanism of *P. falciparum *resistance is unique to the inverted form or common across forms. In a confusing turn of nomenclature, the inverted arrangement of 2La is actually ancestral, and the standard form is derived [8]. Thus, it is formally possible that the monophyletic inversion event leading to the "standard" chromosome (2La+/+) could have captured a susceptible allele from the PRI. Another mapping study done in East Africa cannot be compared because it did not report the inversion state of 2La [9].

### Resistance alleles maintained in colonies

Prior linkage mapping [3,4] of *P. falciparum *resistance loci used F1 or F2 pedigrees that were only a generation or two removed from the wild, while the current study uses pedigrees generated from a colony established several years earlier. It is important to emphasize the importance of detecting resistance loci in a natural population versus observation in a colony, where selection and genetic drift can have a strong influence on the prevalence of susceptibility/resistance alleles. Repeated observations in pedigrees derived from the natural mosquito population [4] is a reliable proxy for prevalence of resistance/susceptibility in the field. In contrast, breeding in a colony can amplify or diminish the prevalence of the relevant alleles. Thus, it is only in light of the previous West African results [3,4], as well as a consistent observation from an independent effort [9], that it can be concluded that the chromosome 2L PRI is indeed a common resistance mechanism in the Kenyan field population.

Detection of *P. falciparum *resistance alleles in a colony-derived pedigree demonstrates that despite the large loss of genetic diversity that accompanies colonization [17], and the absence of any putative parasite-imposed selective pressure for many generations, alleles for resistance and susceptibility to parasite infection can still segregate within a colony. The ability of resistant and susceptible alleles to persist in a colony setting is advantageous for future mechanistic studies of natural genetic resistance factors.

### Parasite strain transcendence

The current mapping study allowed testing for effects across parasite isolates within a mosquito pedigree. Detection of linkage in a pedigree in which different mosquitoes were fed on multiple distinct parasite isolates is consistent with the fact that the allele for resistance in the EA_Pfin1 mapping pedigree controls an effect that transcends parasite strain. This is based on the assumption of non-relatedness between parasite isolates, an assumption which is consistent with the previous analysis of the same infected pedigrees [6]. Parasite strain transcendence or independence is evident from the genetic mapping in West Africa [4], in which multiple independent isofemale pedigrees over multiple years, each fed on blood from a different gametocyte carrier, nevertheless yielded loci in the same 2La genomic region.

## Conclusion

Here it is established that the genomic region of *A. gambiae *chromosome 2L, containing strong *Plasmodium*-resistance loci originally identified in pedigrees from a West Africa population of vector mosquitoes, is not a geographically local phenomenon. At the level of resolution of the genetic mapping, the same genetic factor also exists in East African *A. gambiae*. The intriguing prospect of shared *P. falciparum *resistance loci across Africa makes it a promising approach to consider taking advantage of widespread natural *Plasmodium *resistance mechanisms for the control of malaria transmission  [18,19].

## Authors' contributions

This work was a collaborative effort involving all authors. LL initiated and infected the Kenyan pedigrees and JK provided DNA samples from them. MMR performed genotyping assays and statistical analysis. KM performed statistical analyses. IS and AX typed the pedigree yielding a linkage signal for the 2La inversion. MMR, KM, and KV discussed analysis of results and interpreted their meaning. Both MMR and KM wrote initial drafts of the manuscript. The final draft of the ms was read and approved by all authors.
